# Spectral absorption control of femtosecond laser-treated metals and application in solar-thermal devices

**DOI:** 10.1038/s41377-020-0242-y

**Published:** 2020-02-04

**Authors:** Sohail A. Jalil, Bo Lai, Mohamed ElKabbash, Jihua Zhang, Erik M. Garcell, Subhash Singh, Chunlei Guo

**Affiliations:** The Institute of Optics, University of Rochester, Rochester, NY 14627 USA

**Keywords:** Optics and photonics, Optical materials and structures

## Abstract

Direct femtosecond (fs) laser processing is a maskless fabrication technique that can effectively modify the optical, electrical, mechanical, and tribological properties of materials for a wide range of potential applications. However, the eventual implementation of fs-laser-treated surfaces in actual devices remains challenging because it is difficult to precisely control the surface properties. Previous studies of the morphological control of fs-laser-processed surfaces mostly focused on enhancing the uniformity of periodic microstructures. Here, guided by the plasmon hybridisation model, we control the morphology of surface nanostructures to obtain more control over spectral light absorption. We experimentally demonstrate spectral control of a variety of metals [copper (Cu), aluminium (Al), steel and tungsten (W)], resulting in the creation of broadband light absorbers and selective solar absorbers (SSAs). For the first time, we demonstrate that fs-laser-produced surfaces can be used as high-temperature SSAs. We show that a tungsten selective solar absorber (W-SSA) exhibits excellent performance as a high-temperature solar receiver. When integrated into a solar thermoelectric generation (TEG) device, W-SSA provides a 130% increase in solar TEG efficiency compared to untreated W, which is commonly used as an intrinsic selective light absorber.

## Materials processing: laser modified metals

Femtosecond-laser processing of materials has many potential applications including high-efficiency incandescent lamps and solar-thermal receivers. A longstanding challenge is the ability to control the emerging properties of the treated surfaces. A research team at Chunlei Guo’s lab from the Institute of Optics at the University of Rochester showed that the spectral absorption properties of treated metals can be controlled systematically by controlling the laser processing parameters. They showed that the formed structures act as interacting nano-antennas which absorbs light based on the excitation of electromagnetic resonances. As a direct application, they converted a Tungsten surface to a selective solar-absorber and used it to operate a thermoelectric generator which resulted in a 130% increase in efficiency compared to an untreated tungsten receiver.

## Introduction

Direct femtosecond (fs) laser processing is a cost-effective, maskless and scalable fabrication technique that has been widely used to effectively modify the optical, electrical, mechanical and tribological properties of materials^1–3^. The creation of random structures by fs-laser pulses exhibits desirable features for material functionalisation, e.g., perfect light absorption, superhydrophobicity and superhydrophilicity, with many potential applications in biomedical, environmental, and energy fields^1–8^. However, the ability to design and engineer these properties is very limited as they originate from random structures with random sizes, geometries, and densities.

To provide more control over laser-induced surface structuring, past studies have mainly focused on structural regularity, mostly on the microscale. Of interest, one-dimensional (1D) femtosecond laser-induced periodic surface structures (fs-LIPSSs) with subwavelength periodicity that can be realised on a wide range of materials have attracted considerable attention^1,2^. Different techniques have been employed to address the spatial uniformity of fs-LIPSSs, for instance, positive and negative feedback mechanisms on titanium substrates^9^, chemical-assisted fs-laser-treatment^10^, high-uniformity fs-LIPSSs creation via temporally delayed multi-pulse irradiation^11,12^, non-ablative fs-laser structuring technique^13^, or self-organisation from metal-assisted pattern formation on glass^14^. While these studies showed the ability to produce more regular surface features, they did not demonstrate the ability to design the surface properties, which is crucial at the device engineering and application level.

In this work, we control the morphology of surface nanostructures guided by the plasmon resonance size effect and the plasmon hybridisation model^15–18^, which allows us to control the optical properties of fs-laser-treated metals. We show that the optical absorption of fs-laser-treated surfaces can be altered by tuning the size and density of randomly distributed nanostructures by considering the convolution of the hybridised plasmonic modes of the random structures. Since the particle size and density can be controlled by modifying the fs-laser processing parameters, we use the hybridisation model to guide our fabrication of fs-laser-treated light absorbers. As an application, we create selective solar absorbers (SSAs) and broadband absorbers (BBAs) on Al, Cu, steel, and W. We show that for solar-thermal applications, W-based SSA has the highest solar absorption efficiency. We compare the performance of untreated W (commonly used as a solar absorber) and fs-laser-treated W, as a receiver for a solar thermoelectric generator and show that W-SSA provides a 130% increase in the thermoelectric generation efficiency.

## Results

### Numerical analyses of hybridised metallic surface nanostructures

**Fig. 1:**
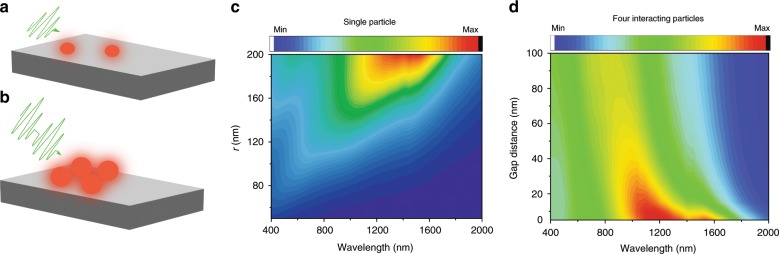
Controlling the absorption spectral range of fs-laser-treated metals. **a** Using low pulse number and lower fluence, the created surface nanostructures are smaller in size and less dense than the surface nanostructures formed **b** using high pulse number and higher fluence. Under optical excitation, larger and denser particles hybridise, and their near-field couples leading to a shift in the plasmon resonance. **c** The calculated absorption for a single W nanoparticle as a function of the particle size highlighting the relevance of the size effect on the broadness of the measured plasmon resonance. **d** The calculated absorption of four hybridized W nanoparticles with a radius of 100 nm as a function of the gap distance between the nanoparticles. A smaller gap leads to stronger hybridisation and redshifts the plasmon resonance peak to higher wavelengths. Both the size and hybridisation effects broaden the measured resonance for randomly distributed nano-surface structures.

The goal of this work is to provide fabrication design tools to control the absorption spectral range of fs-laser-treated metallic substrates. For metallic surface structures with random sizes and distribution, the absorption spectral range depends on two main effects. First, the absorption of a nanostructure broadens and redshifts as a function of the nanoparticle size when the particle size exceeds the quasi-static approximation limit^19,20^. In addition, as the size and density of nanoparticles increase, their individual resonances hybridise due to the so-called plasmon hybridisation effect. The plasmon hybridisation model draws an analogy between surface plasmon coupling and hybridisation of atomic orbitals^15–18^, where coupled metallic (plasmonic) nanoantenna create bonding and antibonding modes that occur at lower and higher frequencies, respectively, compared to the original plasmon resonance^18^. The antibonding, high energy mode is a dark mode and cannot be easily excited directly by an external field^18^. The bonding mode is a bright mode that occurs at a lower energy and thus redshifts the measured plasmon resonance^18^. For an ensemble of metallic nanostructures, increasing the size and density of the nanostructures decreases the interparticle distance, hence increasing the hybridisation likelihood and strength^21^ (see Fig. 1a, b). Consequently, increasing the size and density of randomly distributed plasmonic nanostructures significantly broadens the convoluted plasmon resonances and redshifts the overall plasmon resonance^17^. Figure 1c shows the finite-difference time-domain calculation of the absorption for an individual W nanoparticle. Clearly, the plasmon resonance peak redshifts as the particle’s radius increases. Figure 1d shows the calculated absorption for four hybridised nanoparticles with a fixed radius of 100 nm. As the gap distance between the nanoparticles decreases, the plasmon resonance significantly redshifts. The resulting absorption spectrum of randomly distributed nanoparticles is a convolution of all the absorption bands corresponding to the different plasmon bonding states^17,21^. Because the plasmon resonance of most metals lies in the near-UV region (300–500 nm range), it is possible to create a plasmon-based selective solar absorber that covers the solar spectrum (~300–2500 nm) if we control the average interparticle distance using the relative size and density of the plasmonic nanostructures^17^ (see Supplementary information, Figs. S1 and S2 for more details on the absorption from hybridised W nanoparticles).

For fs-laser-treated surfaces, we obtain a distribution of nanoparticle sizes. According to our analyses shown in Fig. 1c, d, increasing the size and density of the nanoparticles will broaden the absorption spectral range due to excitation of higher order resonances and hybridisation between the excited resonances. Importantly, the size and density of randomly distributed surface structures depend on the number of pulses and laser fluence^1,22^. For a low pulse number and low fluence, the processed surfaces form nanostructures with lower density and smaller size, similar to the surface morphology shown in Fig. 1a. However, at a high pulse number and/or high fluence, the size and density of the formed nanostructures increases, similar to the surface morphology shown in Fig. 1b. Consequently, guided by the plasmon absorption and hybridisation theory, we can intelligently control the fs-laser fabrication parameters to control the hybridisation strength of the surface nanostructures and, as a result, the absorption spectral range.

### Creation of selective and broad band light absorbers with fs-laser ablation

Control over the absorption spectral range of surfaces is of major importance for a wide range of applications, such as selective solar absorbers, thermal emitters, structural colouring^23,24^, water condensation^25^ and daytime and night-time radiative cooling^25^. In particular, for a solar-thermal energy absorber operating at high temperature, the absorber should be an SSA since the main cooling mechanism is thermal radiation, and the light absorber temperature is given by $T_{{\mathrm{absorber}}} \approx \left( {T_{{\mathrm{amb}}}^4 + \alpha _{{\mathrm{solar}}}I_{{\mathrm{solar}}}/\sigma \varepsilon _{{\mathrm{IR}}}} \right)^{1/4}$, where *T*_absorber_ and *T*_amb_ are the absorber and ambient temperatures, respectively, *α*_solar_ is the receiver’s solar absorptance, *I*_solar_ is the incident solar radiation, *ε*_IR_ is the blackbody emittance at the operation temperature, and σ is the Stefan-Boltzmann constant^26^. Accordingly, an ideal solar light absorber has nearly 100% absorbance within the solar spectrum and negligible thermal emittance within the blackbody radiation spectral range at mid-to-high temperatures (100–500 °C), i.e., an SSA. SSAs can thus maximise the temperature of solar absorbers and increase the efficiency of a heat engine driven by solar radiation. In past studies, several materials and structures have been introduced as SSAs, including intrinsic absorbers, e.g., tungsten^26,27^, semiconductor-metal tandems^28,29^, multilayer metal-dielectric interference stacks^30^, metal-dielectric composites^31^, surface textured metals^32,33^ and photonic crystals^34^, and metamaterial and plasmonic light absorbers^35,36^. Although many of these systems demonstrated high design flexibility for light absorption^26,37^, their stability at elevated temperatures necessary for solar-thermal energy conversion applications is highly questionable. In our study, we fabricate an SSA through our controlled fs-laser processing. More importantly, this is the first demonstration of a fs-laser-treated surface that is stable for high-temperature operation. We also demonstrate a high-temperature solar-thermoelectric generation (STEG) device with our SSA.

To create SSAs, we want to create a relatively narrow band absorber that selectively absorbs the solar spectrum (300–2500 nm). To do so, we need to limit the hybridisation between surface nanostructures by creating smaller nanostructures and lower density by using a lower laser fluence at higher scanning speeds, which effectively reduces the pulse numbers. In our experiment, lower fluence levels (0.3–0.5 J cm^−2^) and higher scanning speeds (1–1.5 mm s^−1^) are used to produce SSAs on Al, steel, Cu, and W. Conversely, to create broadband absorbers, we want to increase the hybridisation between surface nanostructures by producing larger nanostructures with a higher density. To do so, we used a set of higher fluence levels (1.5–3 J cm^−2^) and lower scanning speeds (0.1–0.5 mm s^−1^) for Al-BBA, Steel-BBA, Cu-BBA and W-BBA.

**Fig. 2:**
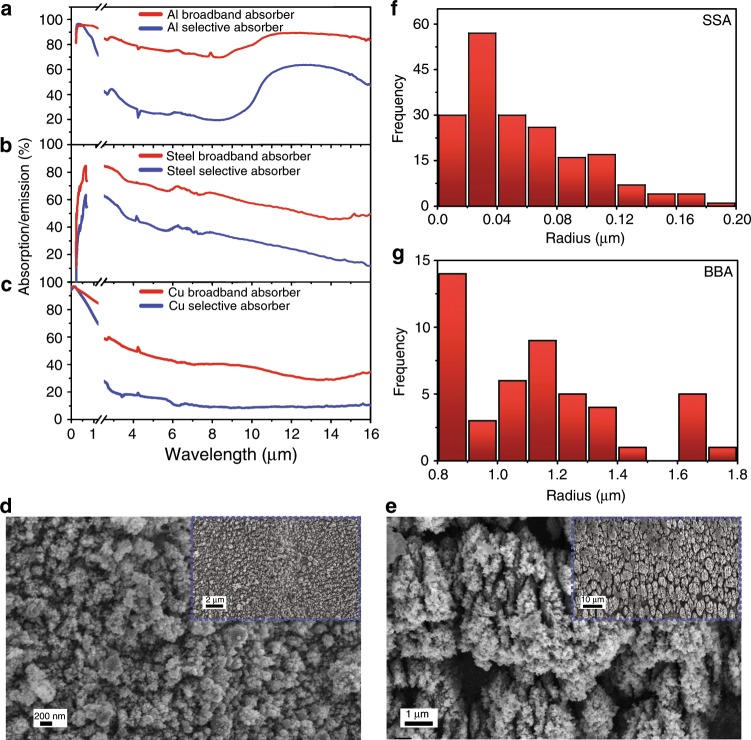
Spectrally selective vs. broadband solar absorbers using fs-laser-treatment: The absorption and emission spectra for SSA and BBA obtained by treating **a** Al, **b** stainless steel and **c** Cu. Cu-SSA shows true spectral selectivity with similar solar absorption and significantly lower thermal emission. SEM images of **d** Cu-SSA and **e** Cu-BBA. The insets show the zoom-out view of Cu-SSA and Cu-BBA. Histograms of the size distribution of the formed surface structures for **f** the Cu-SSA and **g** the Cu-BBA surfaces. The size distribution was obtained using ImageJ.

The spectral absorption/emission of fs-laser processed Al (Fig. 2a), steel (Fig. 2b) and Cu (Fig. 2c) over the visible, NIR and mid-IR ranges is shown. For each metal, we used two laser processing parameters to create a BBA (blue lines) and an SSA (red lines). The fluence used for Al-BBA was 1.5 J cm^−2^, and 3 J cm^−2^ was used for Cu-BBA and Steel-BBA. The scanning speed used for Al-BBA was 0.5 mm s^−1^, and 0.1 mm s^−1^ was used for Cu-BBA and Steel-BBA. The scanning speed used for Al-SSA was 1.5 mm s^−1^, and 1 mm s^−1^ was used for Cu-SSA and Steel-SSA. The fluence for Al-SSA was 0.30 J cm^−2^, and 0.50 J cm^−2^ was used for Cu-SSA and Steel-SSA. Figure 2d, e show scanning electron microscopy (SEM) images of Cu-SSA and Cu-BBA, respectively, which clearly validate the hybridisation model predictions. The insets of Fig. 2d, e shows the low-magnification views of Cu-SSA and Cu-BBA. The average particle size of Cu-SSA is ~0.058 µm, while the average particle size of Cu-BBA is ~1.13 µm. The structure dimensions were determined using ImageJ by considering a 10 × 10 μm^2^ region from an SEM image (see material and methods for details). Using ImageJ, we obtained a histogram of the size distribution of the formed random structures and obtained the average particle size, as shown in Fig. 2f and Fig. 2g, for Cu-SSA and Cu-BBA, respectively. We note here that we are only interested in the particle dimensions in the horizontal plane and not the out-of-plane dimension since the resonances are excited with a normally incident light with horizontal polarisation.

To quantify the performance of the fabricated light absorbers, we first analyse the absorptivity and emissivity of different metals. The spectrally averaged absorptivity $(\bar \alpha )$ of the selective surface is given by^37^:

1 $$\bar \alpha = \frac{1}{I}\mathop {\int}\limits_0^\infty {{\mathrm{d}}\lambda \varepsilon (\lambda )\frac{{{\mathrm{d}}I}}{{{\mathrm{d}}\lambda }}}$$

The emissivity $(\bar \varepsilon )$ is given by:

2 $$\bar \varepsilon = \frac{{\mathop {\int}\limits_0^\infty {{\mathrm{d}}\lambda \varepsilon (\lambda )/\{ \lambda ^5[\exp (hc/\lambda kT) - 1]\} } }}{{\mathop {\int}\limits_0^\infty {{\mathrm{d}}\lambda /\{ \lambda ^5[\exp (hc/\lambda kT) - 1]\} } }}$$

where *I* is the solar intensity, *λ* is the wavelength, *ε*(*λ*) is the spectral emissivity of the selective absorber/emitter, $\frac{{{\mathrm{d}}I}}{{{\mathrm{d}}\lambda }}$ is the spectral light intensity, which corresponds to the air-mass coefficient (AM) 1.5 solar spectrum, *h* is Plank’s constant, *c* is the speed of light, *k* is the Boltzmann constant, and T is the absorber temperature, here, taken as 200 °C. The solar absorber efficiency *η*_abs_ at a given operating temperature for the solar absorber $T_{{\mathrm{abs}}}^4$, and solar concentration *C*, assuming only radiative losses is given by:

3 $$\eta _{{\mathrm{abs}}} = \bar \alpha - \frac{{\bar \varepsilon \sigma (T_{{\mathrm{abs}}}^4 - T_{{\mathrm{amb}}}^4)}}{{CI_{{\mathrm{solar}}}}}$$

where *I*_solar_ is the solar intensity and is ~1000 W m^−2^.

The calculated solar absorptance and emissivity for the BBA and SSA absorbers at *T* = 200 °C, as well as the corresponding *η*_abs_ in percentage for *C* = 1, are shown in Table 1.

**Table 1:** Calculated solar absorptance, emissivity, and solar receiver efficiency for BBAs and SSAs based on the fs-laser-treatment of Al, steel, Cu, and W.

Metal	$\bar \alpha$ (BBA)	$\bar \varepsilon$ (BBA)	*η*_*abs*_ (*BBA*)	$\bar \alpha$ (SSA)	$\bar \varepsilon$ (SSA)	*η*_*abs*_ (*SSA*)
Al	0.94	0.76	−92%	0.81	0.38	−13.5%
Steel	0.90	0.63	−64%	0.60	0.33	−22%
Cu	0.80	0.46	−33%	0.70	0.2	19%
W	0.88	0.82	−111%	0.92	0.18	45%

Although SSAs consistently have lower $\bar \alpha$, they have higher *η*_abs_. One of the by-products of fs-laser processing of metals is the formation of metal oxides, which limits the possibility of having low $\bar \varepsilon$, even after optimising the laser processing parameters. While it is possible to control the absorption spectral range of Al, a strong absorption peak is produced for wavelengths >8 µm due to the phonon-polariton absorption of the formed Al_2_O_3_. On the other hand, stainless steel forms broad absorption with limited control over its spectral range, and it is difficult to create an SSA using steel. This is likely due to the nonzero extinction coefficient of iron oxides (Fe_3_O_4_ and Fe_2_O_3_) over the entire visible, NIR, and IR ranges (see Supplementary information, Fig. S3). Only Cu produces a positive *η*_abs_; i.e., it is the only metal capable of reaching temperatures higher than the operating temperature (*T* = 200 °C) by only absorbing incident solar radiation at *C* = 1 if cooling is solely due to thermal radiation. Cu, however, can form oxides that have negligible absorption over the wavelength ranges of interest^38^. Therefore, Cu is not a proper choice for high-temperature applications due to its low oxidation temperature^39^ and relatively low melting point (~1000 °C), which could be reduced even further when considering that the absorption in fs-laser-treated metals is due to nanostructures^40^, that have a lower melting point than their bulk counterpart^41^.

**Fig. 3:**
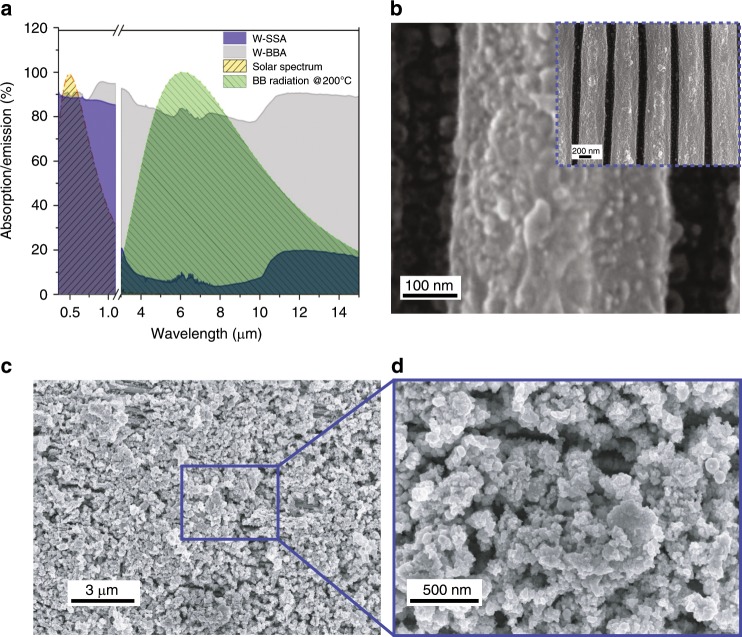
Tungsten as an ideal SSA and BBA. **a** The measured absorption/emission of W spectrally selective absorber and broadband absorber as well as the AM 1.5 solar and radiation spectra of a blackbody (BB) at 200 °C. **b** SEM images of W-SSA showing nanostructure-covered fs-LIPSSs at a fluence of 0.30 J cm^−2^. The inset shows a zoom-out view of the nanostructure-covered fs-LIPSSs. **c** SEM images of microstructures formed for BBA at a fluence of 3 J cm^−2^. A magnified SEM image of the BBA surface is shown in **d**.

W was selected as the best candidate for high-temperature SSA since it has a high melting point (3422 °C) and mechanical strength^42^. We show control over the absorption spectral range by controlling the hybridisation of the formed W nanostructures. Figure 3a shows the measured spectral absorption/emission of broadband (black line) and selective (blue line) W absorbers. The broadband W has a solar absorptance of ~0.88 and emissivity of ~0.82 with *η*_abs_(BB) ~ −111% at T = 200 °C (see Table 1). On the other hand, the W-SSA solar absorptance is ~0.92 and the emissivity is ~0.18 with *η*_abs_ SSA ~ 45% at *T* = 200 °C (Table 1). The results shown in Fig. 3a represent two cases of a highly selective solar absorber and an ultrabroadband light absorber. In the supplementary materials, we show that as we vary the laser fluence, we can tune the structure sizes, which tunes the absorption/emission properties of the W surface (Fig. S4), agreeing with the plasmon hybridisation model shown based on the particle distribution in Fig. S5.

Figure 3b shows SEM images of the fs-laser-treated W with a fluence of 0.30 J cm^−2^ and scanning speed of 1 mm s^−1^. The formed periodic line gratings are nanostructure-covered fs-LIPSSs. The formed nanostructure-covered fs-LIPSSs are frequently reported on a wide range of materials and are formed in a direction perpendicular to the polarisation of the incident beam with a period on the order of the incident wavelength^1,2^. It is important to note that although fs-LIPSSs can act as a 1D grating that excites surface waves, the measured absorption is not due to fs-LIPSSs, but is due to the nanostructures formed on top and in-between fs-LIPSSs. This is because light absorption via exciting surface plasmons (SPPs) with a grating is limited to the grating diffraction orders that satisfy the phase matching condition of the SPPs. For the formed grating, the period is ~530 nm, and the phase matching condition is not satisfied for W at any wavelength, as we show in the supplementary information, Fig. S6. Figure 3c, d show SEM images of W-BBA created at a fluence of 3 J cm^−2^ and a scanning speed of 0.1 mm s^−1^, where clusters of large surface structures are formed, which lead to a broader absorption spectrum according to the hybridisation model.

### High-temperature operation of fs-laser-treated SSA

**Fig. 4:**
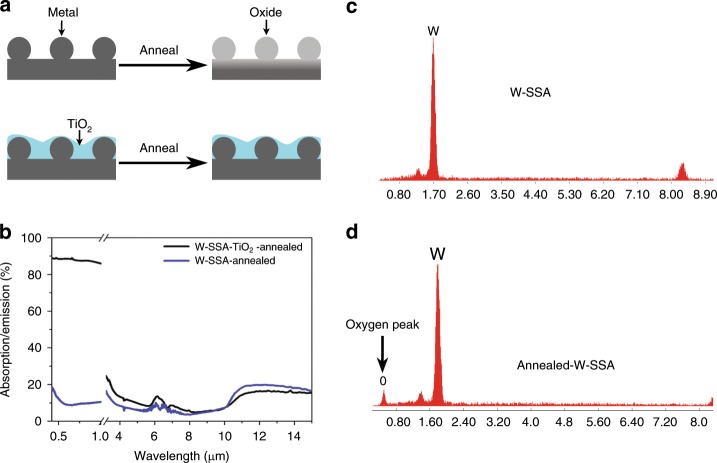
High-temperature operation of W-SSA. **a** Schematics showing the mechanism behind losing the absorption properties of fs-laser-treated metals at high temperatures. The metallic nanostructures are responsible for plasmonic absorption. At high temperatures, the nanostructures oxidise. By adding an oxide layer via thin film deposition, the surface structures are protected, and annealing does not oxidise the surface structures. **b** The measured absorption/emission of the laser-irradiated selective W surface annealed at 500 °C (blue line) and a 200-nm TiO_2_ layer added on laser irradiated selective W surface (black). **c** EDS spectra of W-SSA and **d** annealed W-SSA. An oxide peak appears after annealing at 500 °C.

Although durable and selective, W and other metals can react and form oxides and nitrides at high temperatures if they are placed in an ambient environment. In our experiments, we noticed that the fs-laser-induced light absorption disappears when we heat the metallic light absorber. This is because the surface plasmonic nanostructures, responsible for the observed absorption, turn into oxides at higher temperatures, as shown in Fig. 4a. Since the absorption is due to the excitation of plasmonic resonances, converting a metal to an oxide destroys the surface absorption. To ensure that W survives high temperatures, we deposit a 200-nm-thick TiO_2_ thin film by electron beam evaporation (~0.1 nm s^−1^). By adding a protective oxide layer, the surface nanostructures are no longer exposed to oxygen and retain their metallic properties even at elevated temperatures, as shown schematically in Fig. 4a. We choose TiO_2_, as opposed to Al_2_O_3_ or SiO_2_, as it has low spectral emission for wavelengths <10 µm, i.e., away from the blackbody radiation wavelength range of interest.

To test the performance of TiO_2_-coated W at high temperatures, we annealed TiO_2_-coated W and uncoated W-SSA at 500 °C for ten minutes. As shown in Fig. 4b, the TiO_2_-coated W-SSA survived the annealing process, while the uncoated W-SSA lost its absorption properties. By performing energy-dispersive spectroscopy (EDS) on uncoated W before (Fig. 4c) and after (Fig. 4d) annealing, we can see that an oxide peak appears post-annealing. A cross-sectional SEM image of W-SSA shows the deposited TiO_2_ film (see Supplementary information, Fig. S7). We note that the disappearance of the strong light absorption associated with the surface structures indicates that light trapping effects do not contribute significantly to the observed light absorption. This is because after annealing, the formed structures persist (see Supplementary information, Fig. S8), while the measured absorption is similar to that of an untreated surface. Because the absorption is due to plasmonic resonances, the absorption disappears when the metallic surface structures oxidise.

### Solar thermoelectric generation using fs-laser-treated W

**Fig. 5:**
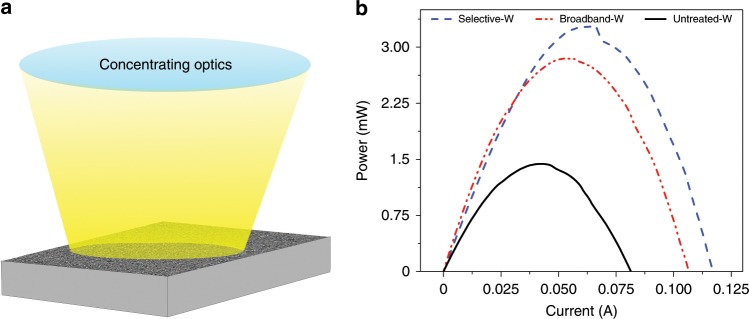
Enhanced solar thermoelectric generation. **a** A schematic illustration of the STEG experiment where we used concentrating optics to obtain a high solar concentration on a TEG with a solar-thermal receiver consisting of W-SSA, W-BBA and untreated-W. **b** The measured STEG output power vs. current for the three STEGs showing maximum power output for W-SSA.

To present a practical device application of our plasmon hybridisation-based fabrication method, we tested the TEG efficiency of three thermoelectric generators with a solar thermal receiver consisting of W-SSA, W-BBA and untreated-W. We note that W is used as an SSA since it has good absorption within the solar spectrum and low emissivity in the blackbody radiation range. As shown schematically in Fig. 5a, to obtain high temperatures, we used an optical concentration of *C* = 40. The maximum temperatures reached by the untreated W, W-BBA, and W-SSA were 118 °C, 155 °C and 178 °C, respectively. We note that due to the HVAC system in the lab, the forced convection current leads to a high convection coefficient, which can reach up to 200 ${\mathrm{Wm}}^{ - 2}{\mathrm{K}}^{ - 1}$. Figure 5b shows the measured STEG output power vs. the current for the three systems. The maximum output power of W-SSA is ~130% and 15% higher than that of untreated W and W-BBA.

## Discussion

In conclusion, we used the plasmon hybridisation model to explain the absorption spectral range of fs-laser-treated metals. We were able to control the surface morphology by adjusting the fs-laser parameters to create selective solar light absorbers and broadband light absorbers on different metals. We showed that metals with oxides that do not absorb significantly in the blackbody radiation wavelength range, e.g., Cu and W, are excellent candidates for selective solar absorption. We further investigated the performance of the fs-laser-treated SSAs at high temperatures and showed that W provides the best performance. To have fs-laser-treated W operate as a high-temperature SSA in ambient environments, the W surface must be sealed with a dielectric thin film. We chose TiO_2_ as it has limited absorption within the blackbody radiation wavelength range of interest. W absorbers coated with TiO_2_ were shown to withstand annealing at 500 °C without any noticeable degradation in their absorption properties. We showed that the maximum power output is obtained from a thermoelectric generator using W-SSA as the solar receiver, which improved the output power efficiency by ~130% compared to using untreated W as the solar receiver. For the first time, we produce fs-laser-treated surfaces that can act as high-temperature absorbers for enhanced thermoelectric generation efficiency.

## Materials and methods

### Laser fabrication setup

The laser used in our experiments is a Ti: sapphire fs-laser amplifier, which delivers horizontally polarised pulse trains at a repetition rate of 1 kHz with a central wavelength of *λ* = 800 nm and a pulse duration of *τ* = 30 fs. The maximum pulse energy delivered by the laser system is 7 mJ. The laser was focused by a lens with a focal length of 20 cm and incident at normal incidence. Bulk circular disks of W, Al, Cu and stainless steel (obtained from Alfa-Aesar with 99.99% purity) were used as target materials due to their high mechanical strength at high temperatures. The samples were mounted on a computerised XYZ precision stage and translated at different speeds for broadband and selective absorbers.

### Simulations

The simulations were performed using commercially available finite-difference time-domain software from Lumerical®. For all simulations, we calculated the absorption inside W nanoparticles as a function of the particle size (Fig. 1c), or the gap distance (Fig. 1d, Figs. S1 and S2).

### Determination of particle radii from SEM images

In our work, we are interested in resonances occurring due to the excitation of dipoles oscillating parallel to the surface, i.e., the x–y plane. To obtain the dimensions in the x–y plane of the nanoparticles, we used ImageJ, which is conventionally used to measure particle dimensions. We first select an evenly illuminated region from the SEM surface image and then apply a bandpass filter to flatten the image. Afterwards, we apply a threshold to the image by adjusting the background and outline the particles. After obtaining the area distribution of the outlined particles, we can directly calculate the particle radius distribution.

### Physical vapour deposition of TiO_2_

To create an SSA that operates at high temperature, a 200 nm TiO_2_ film is deposited by an electron beam evaporator on the processed metals. As discussed above, the TiO_2_ film acts as a protective layer against oxidation of the surface nanostructures, which destroys the absorption properties of the ablated surfaces. TiO_2_ pellets were purchased from Kurt J. Lesker® with 99.9% purity and evaporated via electron beam evaporation at 0.1 nm sec^−1^.

### Annealing

To check the stability of the solar absorber, we annealed the samples in an oven at 500 °C for ten minutes.

### Spectral and surface characterisation

To characterise the spectral reflectance/scattering, we measured the total hemispherical optical reflection of the samples using an ultraviolet-visible (UV) PerkinElmer Lambda 900 spectrophotometer and Fourier transform infrared spectroscopy (FTIR), Bruker IFS 66/S FTIR spectrometer, each equipped with an integrating sphere. The surface morphology was analysed by SEM, and EDS was performed to study the presence of oxides and nitrides. Ion-beam milling was utilised for the cross-sectional view of the oxide layer.

### TEG measurements

Commercial Bi-Te-based TEGs with a size of 18 × 21 mm were purchased from TEGpro^tm^ (module of TE-MOD-1W2V-21S). Bi-Te-based TEG power modules can operate at temperatures as high as 230 °C continuously. A solar simulator with an AM 1.5 airmass filter was used. A plano-convex lens with a 250 mm focal length and 150 mm diameter was mounted at the output port of the solar simulator to enhance the optical concentration. Power was measured using a Keithley-2400 source metre by using an open circuit voltage and sweeping the voltage down to 0 while measuring the current. The receiver temperature was monitored using a thermocouple.

## Supplementary information

Supplementary Informantion
