# Supplementary material for: Spectral absorption control of femtosecond laser-treated metals and application in solar-thermal devices
Source: Light Sci Appl. 2020 Feb 4;9:14. doi: 10.1038/s41377-020-0242-y (PMC7000826; doi:10.1038/s41377-020-0242-y)
Supplement: Supplementary file 1 — Supplementary Informantion [file 41377_2020_242_MOESM1_ESM.docx]

Supplementary information for

# Spectral absorption control of femtosecond laser-treated metals and application in solar-thermal devices

Sohail A. Jalil^1^, Bo Lai^1^, Mohamed Elkabbash^1^, Jihua Zhang^1^, Erik M. Garcell^1^, Subhash Singh^1^ and Chunlei Guo^1^

^1^The Institute of Optics, University of Rochester, Rochester, NY 14627, USA

Correspondence: Mohamed ElKabbash ([*melkabba@ur.rochester.edu*](mailto:melkabba@ur.rochester.edu)*);* Chunlei Guo ([*guo@optics.rochester.edu*](mailto:guo@optics.rochester.edu))

These authors contributed equally: Sohail A. Jalil, Bo Lai

**FDTD calculation of three hybridizing nanoparticles**


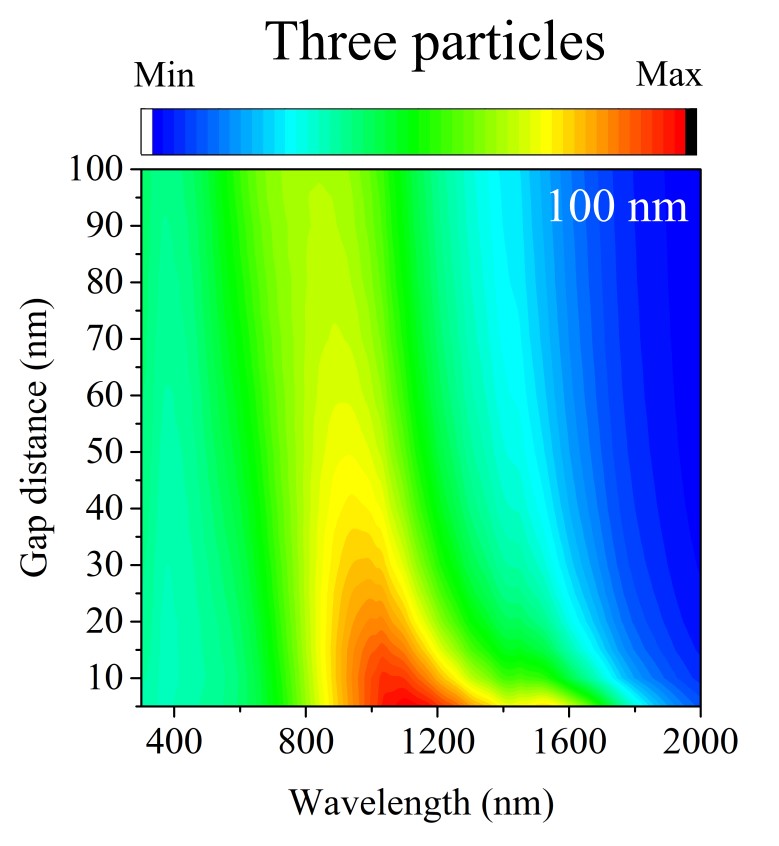


**Figure S1.** The effect of hybridization on the plasmon resonance depends on the number of nanoparticles. The calculated absorption from three particles with *r* = 100 nm is shown here. Compared to the hybridization from four particles shown in the main manuscript [Fig. 1d], it is clear that the hybridization effect (the spectral shift) increases as the number of hybridizing nanoparticles increases.

**FDTD calculation of three hybridizing nanoparticles with 60 nm and 150 nm radius**


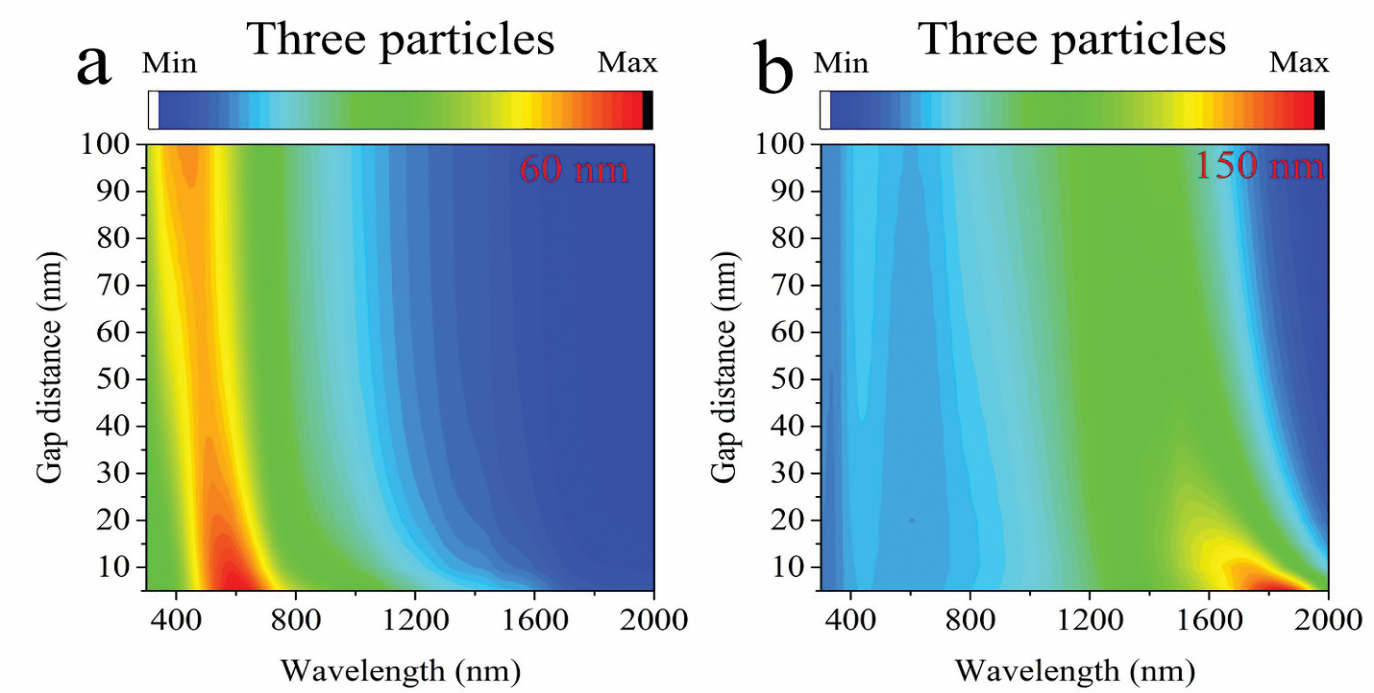


**Figure S2.** The plasmon resonance for three hybridizing nanoparticles with **(a)** *r* = 60 nm, and **(b)** *r* = 150 nm. The calculations show that hybridization between larger nanoparticles can shift the plasmon resonance from the UV-Vis range to IR wavelengths.

**The real and imaginary components of Al_2_O_3_ and magnetite/iron oxide**
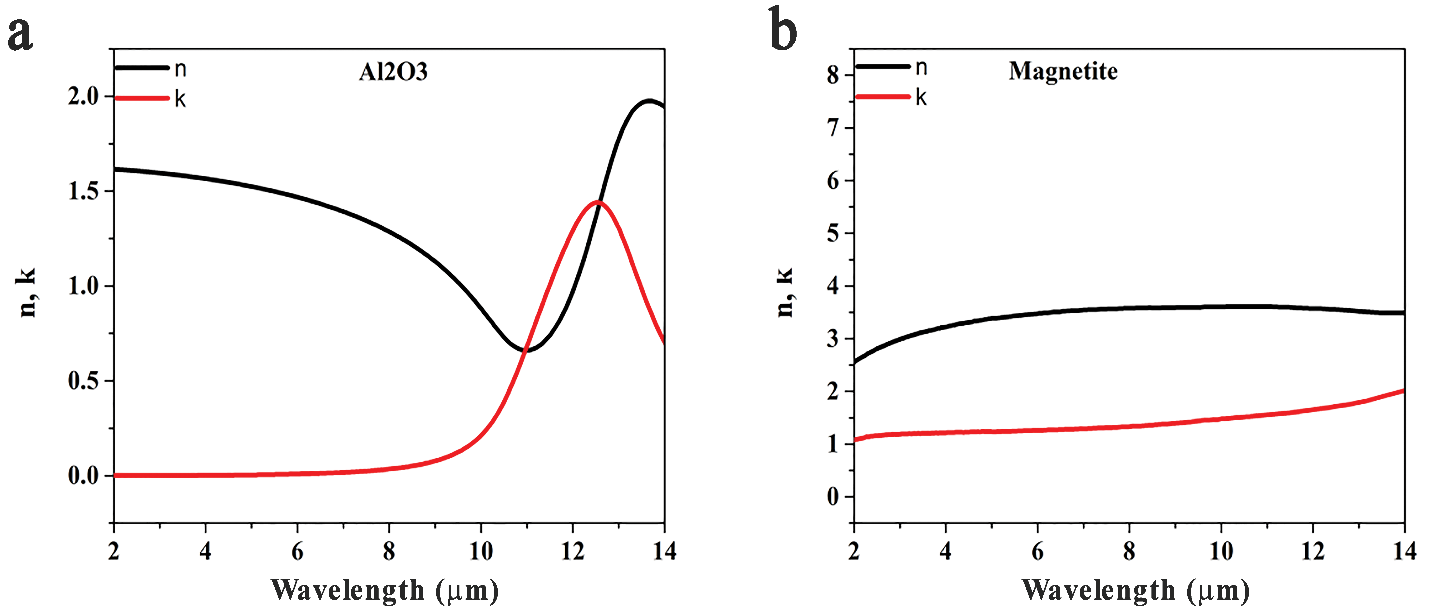


**Figure S3.** The real (*n*) and imaginary (*k*) components of the complex refractive index of **(a)** Aluminum oxide Al_2_O_3_ and an **(b)** iron oxide (magnetite). Materials with *n* and *k* > 1 exhibit strong light absorption. This is realized in Al_2_O_3_ for wavelengths > 8 µm and for magnetite over the entire plotted wavelength range (2 µm to 14 µm).

**The variation of absorptivity as a function of laser fluence**


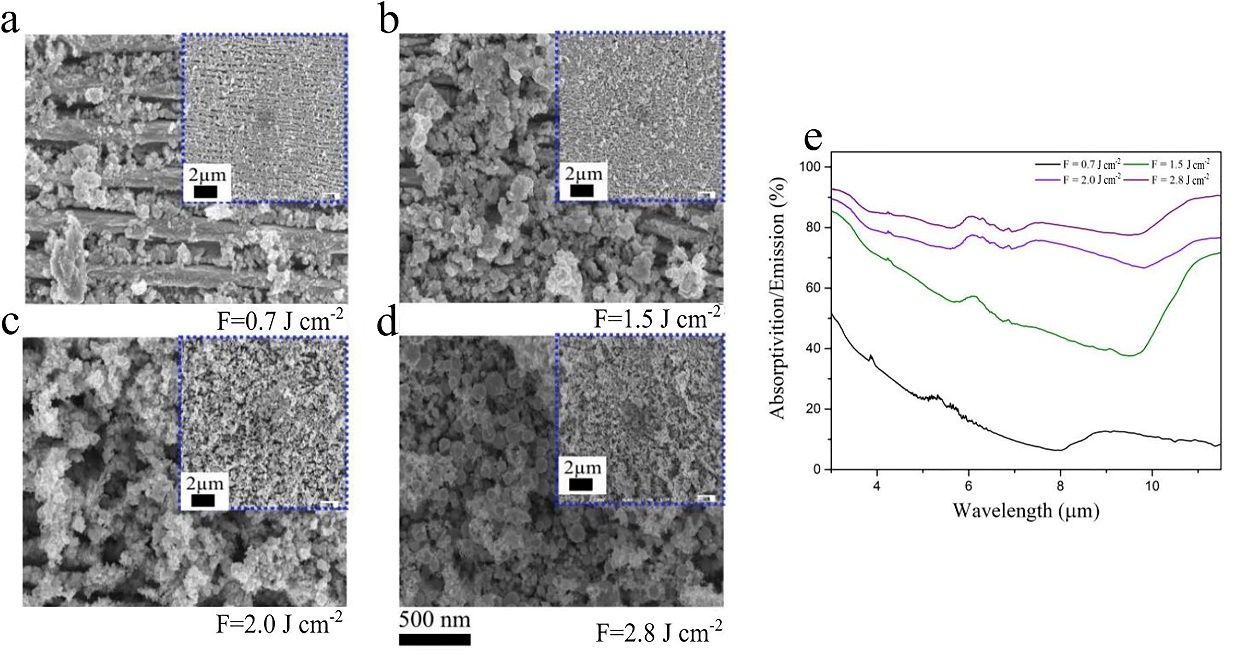


**Figure S4.** Tuning the absorption properties of tungsten by changing the laser fluence for SSA-W and BBA-W. **(a-d)** Shows the SEM images of the W surface structures formed at different laser fluences. Clearly the formation of nanostructures covered fs-LIPSSs [Fig. 3**b** in the main manuscript for *F* = 0.30 J cm^-2^] changes to denser nanostructures covered fs-LIPSSs for **(a)** *F* = 0.7 J cm^-2^ and **(b)** *F* = 1.5 J cm^-2^. Dense micro/nanostructures and clusters are formed for **(c)** *F* = 2 J cm^-2^ and **(d)** *F* = 2.8 J cm^-2^. The inset of Figs. S**4a-d** shows a zoom-out SEM images of the surface. The scale bar of 500 nm is same for Figs. S**4a-d**. **(e)** The corresponding measured absorption/emission showing stronger IR absorption/emission as a function of laser fluence.

**W particle size distribution as a function of the laser fluence**


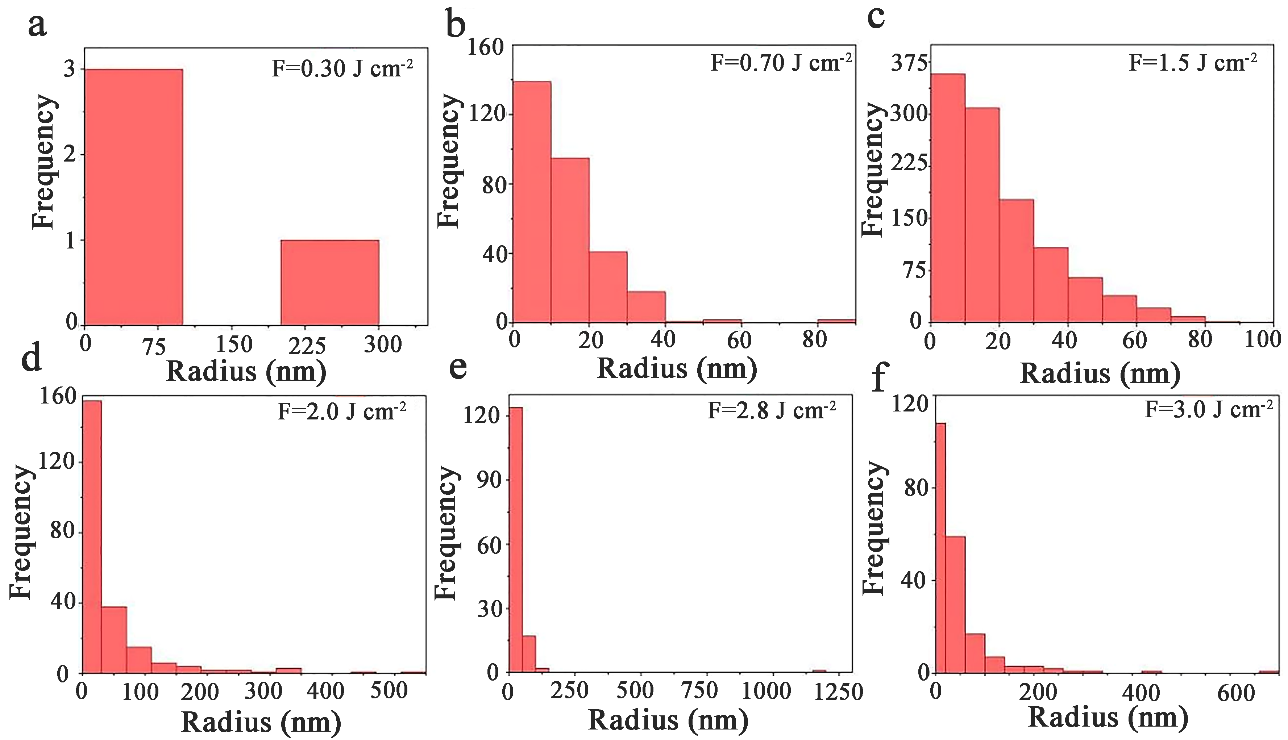


**Figure S5.** Histograms of the size distribution of the formed surface structures at different laser fluences. For **(a)** *F*= 0.30 J cm^-2^, only few nanoparticles are present. Increasing the laser fluence to **(b)** *F*= 0.70 J cm^-2^ and **(c)** *F*= 1.5 J cm^-2^ creates more surface structures, i.e., higher particle density, and average particle size for *F*= 1.5 J cm^-2^. Higher frequency of structures with size > 100 nm are obtained for **(d)** at *F* = 2 J cm^-2^ and **(e)** at *F* = 2.8 J cm^-2^ with microstructures appearing for *F* = 2.8 J cm^-2^. For *F* = 3 J cm^-2^ we observe even higher frequency of large structures. Although the maximum radius is observed for *F*= 2.8 J cm^-2^, the frequency of nanoparticles with radius between 100 nm to 300 nm is higher in case of *F* = 3 J cm^-2^ as compared to *F*= 2.8 J cm^-2^.

**Excluding SPP excitation from fs-LIPSS as a mechanism behind light absorption**


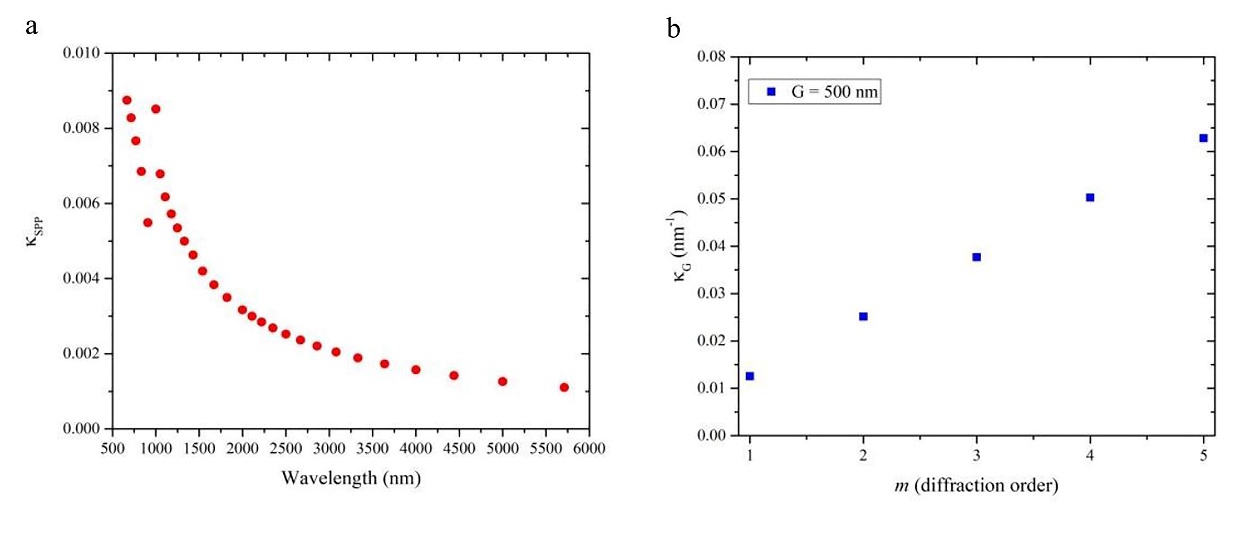


**Figure S6.** **(a)** Shows the calculated SPP momentum at the W/air interface for the wavelength range of interest, $\kappa_{SPP}= \frac{2\pi}{\lambda}\left( \varepsilon/{\varepsilon+1} \right)^{1/2}$, where $\varepsilon$ is the W permittivity. $\kappa_{SPP}$ progressively decrease for longer wavelengths. **(b)** shows the calculated grating momentum $\kappa_{G}=m\frac{2\pi}{G}$, where G is the grating period and is 530 nm in our case, and *m*= $\pm$1, 2,…, is the grating diffraction order. For SPP excited via grating coupling, $\kappa_{SPP}=\kappa_{0} n\sin\theta+\kappa_{G}.$ For normal incidence, as in our case, the excitation of SPP requires $\kappa_{SPP}= \kappa_{G}$ which is not satisfied at any wavelength.

**The cross-sectional view of TiO_2_ deposited on SSA-W**


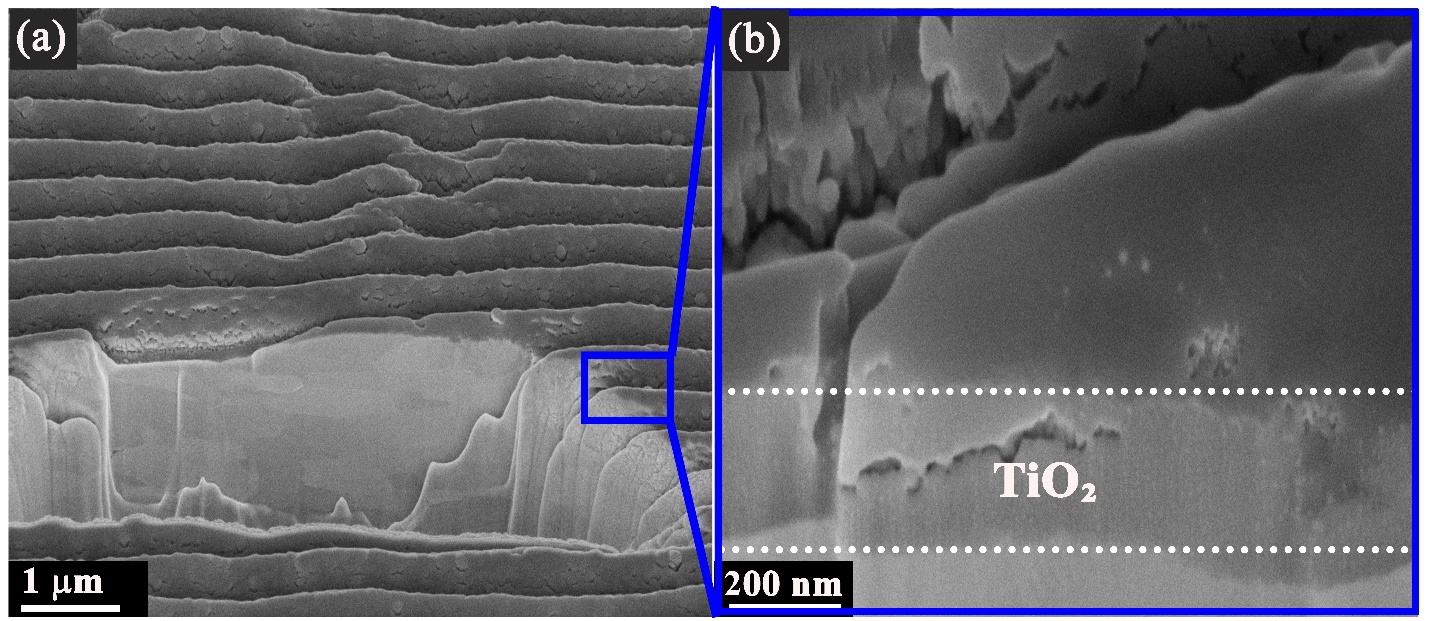


**Figure S7.** Show a cross-sectional SEM image of the W-SSA where we can see the deposited TiO_2_ film. The images were obtained by ion-beam milling the W surface.

**Excluding light-trapping effects as a mechanism for the observed light absorption**


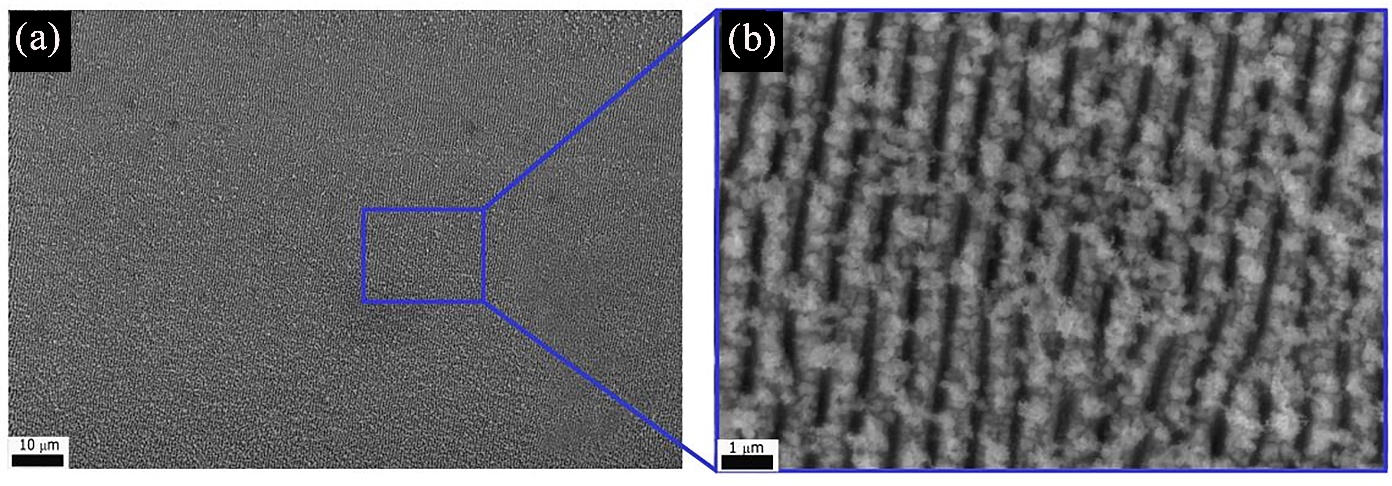


**Figure S8.** **(a)** SEM image (scale bar 10 µm), and **(b)** a Zoom-in SEM image (scale bar 1 µm) of the fs-laser treated W after annealing. The surface structures persist after annealing yet absorption disappears which proves that the metallic nature of the structures is essential for the observed light absorption.
